# Is It What They Eat or How Much They Eat That Matters More in Adults with Food Insecurity in a Wealthy-Country Context?

**DOI:** 10.3390/nu13030851

**Published:** 2021-03-05

**Authors:** Min Gyeong Kang, Sung-Min Yook, Ji-Yun Hwang

**Affiliations:** 1 Nutrition Education Major, Graduate School of Education, Sangmyung University, Seoul 03016, Korea; skekalsrud@naver.com; 2 Department of Foodservice Management and Nutrition, Graduate School, Sangmyung University, Seoul 03016, Korea; smy522@naver.com; 3 Major of Foodservice Management and Nutrition, Sangmyung University, Seoul 03016, Korea

**Keywords:** food insecurity, healthy eating index, dietary adequacy, dietary quality, adults

## Abstract

This study aimed to investigate whether dietary quantity and/or quality differ according to food security levels in the Korean adult population. Dietary adequacy and quality were evaluated by the Korean Dietary Reference Intake and the Korean Healthy Eating Index (KHEI) for adults, respectively, according to three food security levels, i.e., food security, low food security, and very low food security. A total of 7144 Korean adults (aged 19 to 64 years) were selected from cross-sectional data from the 2013–2015 Korean National Health and Nutrition Examination Surveys. The risk of inadequate nutrient intakes of protein (*p trend* = 0.021) and phosphorus (*p trend* = 0.002) increased according to food insecurity levels after adjustment for putative risk factors. The total KHEI scores (*p* < 0.001) as well as scores of having breakfast (*p* < 0.001) were lowest in the very low food security group. Among KHEI components, adults with food insecurity were less likely to get full scores from intakes of mixed grains (*p trend* = 0.016), total fruit (*p trend* = 0.039), fresh fruit (*p trend* = 0.043), and breakfast (*p trend* < 0.001). In addition, food-insecure adults were more likely to get zero score from intakes of fresh fruit (*p trend* = 0.020), milk and dairy products (*p trend* = 0.049), breakfast (*p trend* < 0.001), % of energy from sweets and beverages (*p trend* = 0.002), and total energy (*p trend* = 0.033). In conclusion, food security levels were associated with how much they ate, as well what they ate, in adults in South Korea. These results implied that the diet adequacy as well as moderation and balance could be carefully treated with food assistance or nutrition intervention once nutritional adequacy has mostly been met. In addition, targeted intervention programs tailored to diverse contexts for improving food insecurity may prevent unintended consequences due to easy access to inexpensive obesogenic foods in adults with food insecurity.

## 1. Introduction

Dietary quality has recently become the focus of extensive research interest, given the increased number of diet-related diseases and mortality in developed countries. A prolonged consumption of nutritionally unbalanced meals, such as high intake of sodium and low intakes of whole grains as well as fruits, has been reported to increase the risk of mortality and disability-adjusted life years in many countries [1].

The family members of households with food insecurity are more likely to cope with it by consuming cheap, palatable, high-fat, high-sodium, and high-sugar processed foods [2]. An inverse relationship between food insecurity and nutritional adequacy [3,4,5,6,7,8,9,10,11,12,13] and quality of meals [2,14,15,16,17,18] has been reported in several studies. Food insecurity has been also reported to be associated with eating habits and health behaviors [19,20], which affect physical and mental health [14,21,22,23] in Korea. Therefore, dietary inequality may exacerbate the impact of food insecurity.

Most previous studies have been conducted in low-income or vulnerable populations [2,3,7,15,16,17,18]. Given the fact that food security includes multiple aspects, such as availability, accessibility, and affordability of food, overall dietary quality in addition to nutritional adequacy needs to be evaluated across food security levels in the general adult populations. Furthermore, food insecurity has never been eradicated even in developed countries although abundance in food has been already achieved at the national level. However, information on levels of food security in relation to dietary quality in the general adult population, especially in the wealthy country context, is very limited.

A recent systematic review regarding assessment tools of dietary quality has suggested three aspects of dietary quality: the adequacy of nutrient intake, food variety or diversity, and moderation for intake of foods and food groups or energy and nutrients [24]. According to this previous study, evaluation of moderation among the three aspects was not often included in low- and middle-income countries whereas it has been developed and used in assessment tools such as the healthy eating index (HEI) in the high-income countries, such as the United States (US) [14,15,24,25,26,27,28]. Recently the Korean Healthy Eating Index (KHEI) for adults has been developed in our previous study [29] based on the Korea National Health and Nutrition Examination Survey (KNHANES) VI (2013–2015) and it is currently used in the KNHANES [30].

To the best of our knowledge, there is very little research on relationships between food security levels and overall dietary adequacy as well as quality in the general adult population in a developed-country context, and even no research in South Korea. Therefore, using a nationwide representative sample from the KNHANES, we investigate whether dietary quantity and/or quality differ according to food security levels in the population of general adults in a wealthy country context like South Korea.

## 2. Materials and Methods

### 2.1. Study Design and Subjects

The KNHANES study is an ongoing nationwide population-based cross-sectional study conducted by the Korea Disease Control and Prevention Agency (KDCA) since 1998. Non-institutionalized Koreans are randomly selected using a stratified and multi-stage clustered probability sampling method. Among 11,681 adults aged 19 to 64 years from the KNHANES VI study (2013–2015) with information on household food security levels and HEI data, 705 subjects with implausible daily energy intake (<800 kcal/day or >4000 kcal/day in men, <500 kcal/day or >3500 kcal/day in women; *n* = 705) [31] were excluded. Among the remaining 10,976 subjects, 226 pregnant or lactating women and 1587 with insufficient information on educational level, marital status, household income, height, weight, physical activity, smoking, alcohol drinking, and physical activity were excluded. A total of 7144 subjects were finally eligible for this analysis after further exclusion of 2019 subjects who recently changed their eating habits due to weight control or illness.

A written informed consent was obtained from all participants before the survey. The institutional review board (IRB) of the KDCA approved survey protocols (2013-07CON-03-4C, 2013-12EXP-03-5C, 2015-01-02-6C). Therefore, the additional IRB process was not needed for the current study.

### 2.2. Socio-Demographic Factors

Detailed data on socio-demographic factors and personal characteristics were collected including age, sex, number of household members, education level, marital status, household income level, smoking, alcohol drinking, level of physical activity, height, weight, and disease history. Education status was categorized as ≤elementary school graduate, middle school graduate, high school graduate, and ≥college graduate. Marital status was classified into three categories: married, never married, and separated, widowed, or divorced. Household income levels were classified as low, middle low, middle high, and high. Smoking behavior was classified as non-smoker, former smoker, smoking less than 20 cigarettes/day, and smoking ≥20 cigarettes/day. Alcohol drinking was categorized as non-drinker, <1 drink/month, ≥1 drink–4 drinks/month, and ≥5 drinks/month. The level of physical activity was categorized into four categories: low, middle low, middle high, and high. Body mass index (BMI) was calculated as weight divided by height squared (kg/m^2^). Disease history included having hypertension, dyslipidemia, stroke, myocardial infarction (or angina), diabetes, cancer, kidney failure, or cirrhosis of the liver.

### 2.3. Household Food Security Measurement

In the KNHANES, household food security is measured using an 18-item questionnaire based on the US Household Food Security/Hunger Survey Module (US-HFSSM) since 2012 and is classified into four groups according to scores: food security (0–2 scores), mild food insecurity without hunger (3–7 scores for household with children; 3–5 for household without children), moderate food insecurity with hunger (8–12 for household with children; 6–8 scores for household without children), and severe food insecurity with hunger (13–18 scores for household with children; 9–10 scores for household without children) [32]. In this study, the levels of food security were reclassified as food security (high or marginal food security), low food security (mild food insecurity without hunger), and very low food security (moderate or severe food insecurity with hunger) due to low proportions of moderate and severe stages of food insecurity (0.48% and 0.06%, respectively).

### 2.4. Nutrients Intake Measurement

Dietary intakes were measured by a single 24 h recall based on a weekday’s usual food intake from the participants via face-to-face interviews. Trained and highly skilled interviewers collected information on names of foods and dishes including recipes, brand names of processed foods including names of manufacturers, and amounts of food consumed. After converting all food intakes into each food ingredient intake, daily energy and 14 kinds of nutrients including vitamins and minerals intakes were calculated based on the 8th Korean Food Composition Table (KFCT) by the Rural Development Administration (RDA) of South Korea [33]. Currently, the standard KFCT includes over 3000 key food items frequently consumed in South Korea based on results of KNHANES, and every year about 350 raw and processed food items have been collected, analyzed, and evaluated for data quality under the Association of Official Analytical Chemists (AOAC), Codex Alimentarius (Codex), and Food and Agriculture Organization (FAO)/International Network of Food Data Systems (INFOODS) guidelines [34]. Fatty acid, cholesterol, and dietary fiber were calculated based on the database developed by the KDCA [33]. The adequacy of nutrient intake was assessed using the 2015 Korean Dietary Reference Intakes (KDRIs) [35]. In this study, the adequacy of energy intake was defined as an energy intake meeting 85–115% of the estimated energy requirement, taking into account about a 15% variation of energy intake within individuals based on the total daily energy intake [36]. Macronutrient intakes including carbohydrates, fats, n-3 fatty acids, n-6 fatty acids, and saturated fatty acids were compared according to the recommended percentages of total energy intake by the 2015 KDRIs. The adequate intake was defined as intakes equal to or above recommended nutrient intake (RNI) and below tolerable upper intake level (UL) for protein, vitamin A, vitamin C, thiamine, riboflavin, niacin, calcium, phosphorus, and iron, and was defined as intakes equal to or above the adequate intake (AI) for dietary fiber and potassium. Intakes of cholesterol and sodium were evaluated based on the intake goal.

### 2.5. Korean Healthy Eating Index (KHEI)

The KHEI is a standardized evaluation tool for overall quality of diet by scoring adherence to dietary guidelines for Koreans, which was developed based on data from the KNHANES [29,30]. The KHEI is composed of a total of 14 components (eight for adequacy, three for moderation, and three for balance) and the total score is calculated to be 0–100 points. Among eight adequacy components, five components are given 0–5 points (mixed grain intake, total fruit intake, fresh fruit intake, total vegetable intake, vegetable intake excluding kimchi and pickled vegetables), and 0–10 points for three components (intakes of meat/fish/eggs/legumes, intakes of milk and dairy products, having breakfast). All three moderation components (% of energy from saturated fatty acid, sodium intake, % of energy from sweets and beverages) are given 0–10 points and all of the three balance components (% of energy from carbohydrate, % of energy from fat, energy intake) are given 0–5 points [29].

### 2.6. Statistical Analysis

The stratification variables and weights were considered in the statistical analysis due to the nature of the sampling frame of the KNHANES. Dates were expressed as means with standard error (SE) for continuous variables or number and weighted % for categorical variables. Differences among three groups (food security, low food security, very low food security) were evaluated using a general linear model (Tukey’s test of multiple comparison) or a chi-square test, as appropriate. Multivariable-adjusted logistic regression analysis was conducted to examine the odds ratio (OR) with 95% confidence interval (CI) for nutritional inadequacy and lack of dietary quality (<full score of KHEI components, zero score of KHEI components) across three food security groups. Models were first adjusted for age, sex, survey year, and total energy intake and second for these variables plus number of households, education, household income, marital status, and physical activity. Smoking and alcohol drinking were not included as confounders due to high relation to other confounding factors. The tendency (*p trend*) across the three groups was also evaluated after adjustment for potential confounders. All the analyses were performed using IBM SPSS Statistics 21 (IBM Co., Armonk, NY, USA). The significance level was considered as *p*-value < 0.05.

## 3. Results

### 3.1. Socio-Demographic Characteristics According to Food Security Status

Of the 7144 Korean adults examined, 6605 (92.5%) were considered to be food-secure, 452 (6.3%) were low food-secure, and 87 (1.2%) were very low food-secure (socio-demographic characteristics are presented in Table 1). As levels of food insecurity increased, household size (*p* = 0.004) and income (*p* < 0.001) decreased. Adults with food insecurity were more likely to be female (*p* = 0.047), less educated (*p* < 0.001), single (*p* < 0.001), drinker (*p* = 0.039), and less physically active (*p* = 0.010). However, age, smoking behavior, body mass index, and disease history did not differ significantly between the three groups.

### 3.2. Nutritional Adequacy According to Household Food Security Status 

Korean adults with food insecurity were less likely to meet dietary guidelines recommended by the 2015 KDRIs for protein (*p* < 0.001), thiamin (*p* < 0.001), riboflavin (*p* = 0.009), niacin (*p* = 0.002), vitamin C (*p* = 0.003), calcium (*p* = 0.022), phosphorus (*p* < 0.001), potassium (*p* = 0.009), and iron (*p* = 0.009) (Table 2). The OR for inadequate nutrient intake increased in both low and very low food security groups, respectively, for protein (OR = 1.49, 95% CI = 1.19–1.85 for low food security; OR = 3.06, 95% CI = 1.84–5.08 for very low food security), thiamine (OR = 1.41, 95% CI = 1.05–1.90; OR = 2.61, 95% CI = 1.55–4.39), niacin (OR = 1.24, 95% CI = 1.00–1.54; OR = 2.26, 95% CI = 1.30–3.92), vitamin C (OR = 1.37, 95% CI = 1.07–1.74; OR = 2.17, 95% CI = 1.12–4.21), phosphorus (OR = 1.47, 95% CI = 1.15–1.88; OR = 4.10, 95% CI = 2.51–6.68), potassium (OR = 1.39, 95% CI = 1.08–1.78; OR = 2.38, 95% CI = 1.31–4.32). In addition, the OR for inadequate nutrient intake increased in adults with very low food security for riboflavin (OR = 1.99, 95% CI = 1.14–3.47), calcium (OR = 4.75, 95% CI = 1.48–15.25), and iron (OR = 2.02, 95% CI = 1.24–3.29). After adjusting for age, sex, survey year, and total energy intakes, the OR for inadequate nutrient intake remained significant for vitamin C (OR = 1.38, 95% CI = 1.07–1.78) in low food security and phosphorus (OR = 3.12, 95% CI = 1.07–9.12) in very low food security. However, these trends did not remain after further adjustment for household size, education, marital status, income levels, and physical activity. Except for protein (*p trend* = 0.021) and phosphorus (*p trend* = 0.002), the risk of inadequate nutrient intake did not increase according to food insecurity levels after adjustment for putative risk factors.

### 3.3. Dietary Quality Measured by the KHEI According to Household Food Security Status

**Figure 1:**
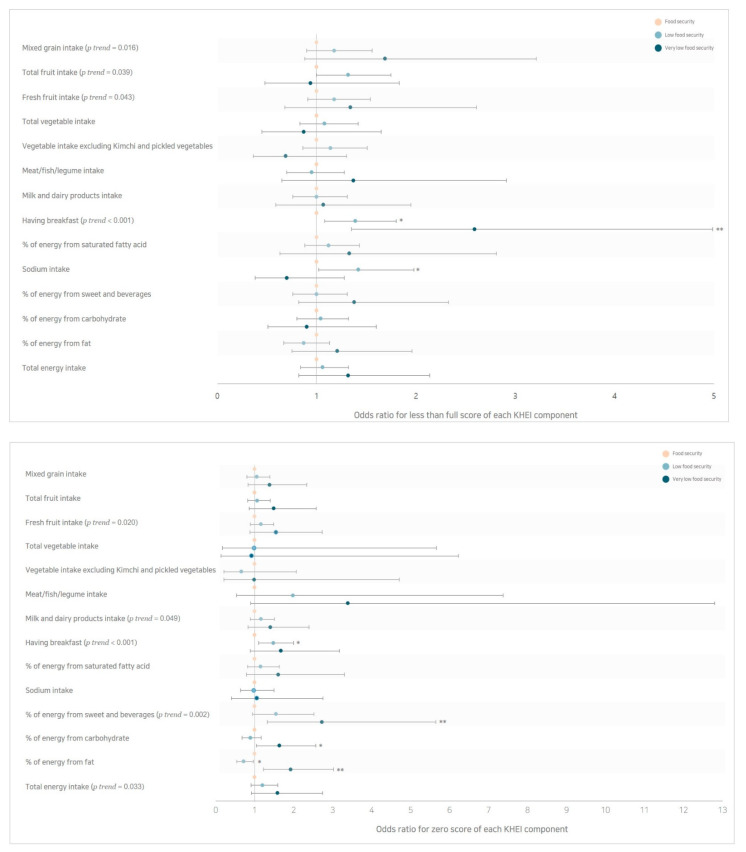
The risk of less than full scores and zero scores according to household food security status. Results based on Model 3 in Table 4 adjusted for age, sex, survey year, total energy intake, household size, education, marital status, household income level, physical activity level. * *p* < 0.05, ** *p* < 0.01. Abbreviations: KHEI, Korean Healthy Eating Index.

The total KHEI scores (*p* < 0.001), as well as scores of having breakfast (*p* < 0.001), were lowest in the very low food security group (Table 3) after controlling for confounding factors. Among KHEI components, adults with food insecurity were less likely to get full scores from intakes of mixed grains (*p trend* = 0.016), total fruit (*p trend* = 0.039), fresh fruit (*p trend* = 0.043), and breakfast (*p trend* < 0.001) and the OR for less than full scores of each KHEI component increased in adults with low food security (OR = 1.39, 95% CI = 1.08–1.80 for having breakfast; OR = 1.42, 95% CI = 1.02–1.98 for sodium intake) and those with very low food security(OR = 2.59, 95% CI = 1.35–4.99 for having breakfast) (Table 4, Figure 1). In addition, food-insecure adults were more likely to get zero scores from intakes of fresh fruit (*p trend* = 0.020), milk and dairy products (*p trend* = 0.049), breakfast (*p trend* < 0.001), % of energy from sweets and beverages (*p trend* = 0.002), and total energy (*p trend* = 0.033). The OR for zero scores of each KHEI component increased in adults with low food security (OR = 1.48, 95% CI = 1.10–2.00 for having breakfast) and those with very low food security (OR = 2.73, 95% CI = 1.32–5.65 for % of energy from sweets and beverages; OR = 1.64, 95% CI = 1.04–2.57 for % of energy from carbohydrate; OR = 1.93, 95% CI = 1.23–3.02 for % of energy from fat). However, the OR for zero scores of % of energy from fat was lowest in adults with low food security (OR = 0.72, 95% CI = 0.54–0.97).

## 4. Discussion

We found a strong and graded association between food security and nutritional adequacy and quality in a Korean adult population. The risk of inadequate nutrient intakes of protein and phosphorus increased as food security decreased. The total KHEI scores were lowest in the very low food security group. Among KHEI components, adults with food insecurity were less likely to get full scores from intakes of mixed grains, total fruit, fresh fruit, and breakfast. Moreover, food-insecure adults were more likely to get zero scores from intakes of fresh fruit, milk and dairy products, breakfast, % of energy from sweets and beverages, and total energy. This association appeared to be independent of other well-known risk factors for nutritional inadequacy and low dietary quality, such as age, sex, household size, education, marital status, household income levels, total energy intake, and physical activity. Our findings suggest that low food security may be an independent risk factor for nutritional inadequacy and lack of dietary quality in Korean adults.

Our result is consistent with previously reported findings [37,38,39,40,41,42]. Food insecurity was related to inadequate intake of vegetables, fruits, meats, and meat substitutes in adult women in northern Jordan [37]. An inverse association between food security and regular breakfast intake was also observed previously in a community-based survey of Korean adults [21]. Breakfast skipping reduced total energy intakes in US adults with food insecurity [38]. Household food insecurity was inversely associated with intakes of fruits and vegetables in the US [41] and Korean [39] adults. Moreover, an inverse association between levels of food security and intakes of milk and dairy products was also observed in adults in Korea [9] and women in northern Jordan [42]. The overall diet quality of adults with very low food security in our study was in line with previously reported studies [5,9,21,37,38,39,40,41,42,43,44,45,46,47,48], which showed reduced consumption of fruits and vegetables, increased consumption of sweets and beverages, and increased % of energy from carbohydrate and fat.

Nonetheless, some studies have also shown inconsistent results. In our study, no differences existed in nutrition adequacy as well as moderation intake of sodium and energy according to food security levels after controlling for confounders, although the tendency of getting zero scores from total energy intake increased as food security decreased. Consumption of sodium increased as total energy intake increased in the US adults with food insecurity [45]. These incompatible findings may be attributable to differences in dietary habits or ethnic differences. Furthermore, an inadequate iron intake was observed in the very low food security group in our study, but the risk of inadequacy did not exist after adjusting for confounding factors. Decreased hemoglobin levels and increased iron binding capacity were observed as food insecurity increased in Korean adults [49]. Persistent iron deficiency leads to hypocytosis or hypochromic anemia. Iron deficiency is also inversely associated with work efficiency, intellectual performance, and resistance to infection [50]. The best food sources of iron are meat, fish and shellfish, and poultry rich in heme irons, and the next best source foods are grains, beans, and dark green vegetables [35]. In our study, the total KHEI scores were lowest in the very low food security group, although no differences in each component except for breakfast intake existed across food security levels. In addition, no differences in overall KHEI scores were observed between adults with food security and low food security. Adequacy or sufficiency of food and nutrient intakes was mainly used to evaluate the quality of diet in the context of developing countries [51]. However, overall dietary quality needs to be carefully considered even though adequacy was almost met, especially in adults with very low food insecurity in the context of developed countries such as South Korea.

Several mechanisms for the effects of food insecurity on lack of dietary adequacy and quality have been proposed. Breakfast skipping increased as food insecurity increased in our study. Lack of knowledge related to diet was known to cause breakfast skipping that leads to insufficient daily energy consumption in households with food [43]. Insufficient daily energy intake, but increased percentages of energy from fat, were due to breakfast skipping in women with household food insecurity [5,47]. Our results suggest that adults with low or very low food security were more likely to get energy from sweets and beverages and were less likely to have fresh fruit and milk and dairy products as shown in previously reported results, showing an inverse association between income levels and intakes of energy from unhealthy desserts such as sugars and sweetened beverages [46]. There exists geographical or regional disparity in food security, so called food deserts. Especially in underprivileged metropolitan areas, excessive supply of high energy-dense foods in households with food insecurity may be attributable to easy access to cheaper, more delicious, and easier to prepare foods than healthy foods [44]. Therefore, policies and programs for food security are thought to be difficult due to the nature of multifaceted aspects of food insecurity, such as income or educational levels, family composition, social safety net, unemployment, and social isolation [46,52,53,54,55,56,57,58]. Despite global economic growth, problems related to food insecurity are not easily resolved. A fundamental solution with a thoroughly designed and tailored approach to promote food security from a macroscopic point of view is needed in order to minimize disparity in diet and health.

Our study had several limitations that should be addressed in future studies. We observed an association between food security and dietary adequacy and quality only in a cross-sectional setting. Therefore, we were not able to determine whether food security level is a cause or consequence of dietary adequacy and quality. Further research is needed to evaluate the causality between levels of food security and dietary adequacy and quality. Furthermore, although the majority of putative risk factors for dietary adequacy and quality were included in the analysis, there may exist potentially unmeasured confounding factors due to the food environment, food assistance, or other correlated factors of having low income. For example, levels of food security as well as dietary adequacy and quality may be influenced by food support, which was not considered in the study. Future research that includes a wide range of environmental and social factors needs to be conducted. Nonetheless, our study had several advantages. Our study used nationally representative data from 2013–2015 KNHANES. Moreover, the KHEI developed for Korean adults was used to evaluate overall dietary quality [29]. Furthermore, household food security was measured using the most accurate tool of the full 18 items based on the US-HFSSM.

## 5. Conclusions

In conclusion, our results suggest that food security levels were associated with how much they ate, as well what they ate, in adults in South Korea. These results implied that the diet adequacy, as well as moderation and balance, could be carefully treated with food assistance or nutrition intervention once nutritional adequacy has mostly been met. In addition, targeted intervention programs tailored to diverse contexts for improving food insecurity may prevent unintended consequences due to easy access to inexpensive obesogenic foods in adults with food insecurity. Especially, the current era of COVID-19 may exacerbate household food insecurity as reported previously in the US [59]. Therefore, further study needs to examine whether the COVID-19 pandemic may worsen existing disparities and whether timely programs such as food assistance may mitigate food insecurity in the Korean population.

## Figures and Tables

**Table 1:** Socio-demographic characteristics of subjects according to household food security status.

Characteristics	Food Security	Low Food Security	Very Low Food Security	*p*-Value
	(*n* = 6605)	(*n* = 452)	(*n* = 87)	
mean ± SE or *n* (weighted %)				
Age, years	41.3 ± 0.2	42.1 ± 0.7	42.6 ± 1.7	0.335
Household size, *n*	3.3 ± 0.02 ^a^	3.4 ± 0.08 ^a^	2.9 ± 0.19 ^b^	0.004
Body mass index, kg/m^2^	23.5 ± 0.1	23.6 ± 0.2	23.6 ± 0.4	0.812
Female, *n*	3830 (58.0)	295 (65.3)	49 (56.3)	0.047
Disease history	1406 (21.3)	114 (25.2)	24 (27.6)	0.249
Education				<0.001
≤Elementary school graduate	716 (10.8)	96 (21.2)	25 (28.7)	
Middle school graduate	640 (9.7)	65 (14.4)	21 (24.1)	
High school graduate	2620 (39.7)	198 (43.8)	27 (31.0)	
≥College graduate	2629 (39.8)	93 (20.6)	14 (16.1)	
Marital status				<0.001
Married	4875 (73.8)	284 (62.8)	36 (41.4)	
Never married	1310 (19.8)	88 (19.5)	25 (28.7)	
Separated, widowed, divorced	420 (6.4)	80 (17.7)	26 (29.9)	
Household income				<0.001
Low	496 (7.5)	132 (29.2)	47 (54.0)	
Middle low	1590 (24.1)	197 (43.6)	23 (26.4)	
Middle high	2160 (32.7)	104 (23.0)	16 (18.4)	
High	2359 (35.7)	19 (4.2)	1 (1.1)	
Smoking behavior				0.346
Nonsmoker	4108 (62.2)	273(60.4)	45 (51.7)	
Former smoker	1121 (17.0)	63(13.9)	13 (14.9)	
<20 cigarettes/day	854 (12.9)	76(16.8)	18 (20.7)	
≥20 cigarettes/day	522 (7.9)	40(8.8)	11 (12.6)	
Alcohol drinking				0.039
Non-drinker	519 (7.9)	49 (10.8)	8 (9.2)	
<1 drink/month	2156 (32.6)	176 (38.9)	32 (36.8)	
≥1 drink–4 drinks/month	1915 (29.0)	114 (25.2)	21 (24.1)	
≥5 drinks/month	2015 (30.5)	113 (25.0)	26 (29.9)	
Physical Activity				0.010
Low	2959 (44.8)	210 (46.5)	50 (57.5)	
Middle Low	1913 (29.0)	153 (33.8)	19 (21.8)	
Middle high	881 (13.3)	36 (8.0)	10 (11.5)	
High	852 (12.9)	53 (11.7)	8 (9.2)	

^a, b^ Different letters indicate the significant statistical difference, same letters indicate no significant difference (*p* < 0.05, Tukey’s test).

**Table 2:** Risk of nutritional inadequacy based on the KDRIs according to household food security status.

	Mean	Median	10th, 90th Percentile	% Meeting Guideline ^1^	Model 1 ^2^		Model 2 ^3^		Model 3 ^4^		*p Trend*
					OR	95% CI	OR	95% CI	OR	95% CI	
Total energy (kcal/day)											0.479
Food security	2084	2012	1167, 3015	35.4 (32.7)	1.0		1.0		1.0		
Low food security	1960	1862	1088, 2925	31.6 (2.0)	1.13	0.89–1.44	1.14	0.90–1.44	1.03	0.80–1.32	
Very low food security	1674	1593	884, 2428	24.1 (0.3)	1.37	0.83–2.26	1.38	0.84–2.29	1.20	0.72–2.00	
				*p* = 0.289							
Carbohydrate (g/day)											0.202
Food security	313	301	180, 455	27.9 (25.8)	1.0		1.0		1.0		
Low food security	300	293	163, 442	27.7 (1.7)	0.95	0.74–1.24	0.92	0.72–1.21	0.84	0.64–1.10	
Very low food security	261	239	138, 415	24.1 (0.3)	1.00	0.57–1.74	0.97	0.57–1.66	0.84	0.49–1.46	
				*p* = 0.936							
Fat (g/day)											0.676
Food security	47	42	15, 81	55.4 (51.2)	1.0		1.0		1.0		
Low food security	43	35	13, 77	53.1 (3.4)	1.05	0.82–1.33	1.01	0.79–1.29	0.87	0.67–1.13	
Very low food security	33	29	8, 63	43.7 (0.5)	1.52	0.94–2.48	1.39	0.87–2.24	1.11	0.69–1.80	
				*p* = 0.253							
N-6 fatty acid (g/day)											0.753
Food security	10.0	8.3	2.7, 18.1	41.9 (38.7)	1.0		1.0		1.0		
Low food security	9.0	7.0	2.4, 16.5	36.1 (2.3)	1.17	0.93–1.48	1.11	0.87–1.41	0.93	0.74–1.20	
Very low food security	7.6	5.0	1.8, 15.9	34.5 (0.4)	1.36	0.80–2.29	1.13	0.68–1.89	0.87	0.52–1.45	
				*p* = 0.220							
N-3 fatty acid (g/day)											0.938
Food security	1.6	1.2	0.4, 3.2	48.3 (44.7)	1.0		1.0		1.0		
Low food security	1.5	1.0	0.3, 3.3	45.1 (2.9)	1.12	0.89–1.40	1.09	0.88–1.37	0.95	0.76–1.20	
Very low food security	1.2	0.8	0.1, 2.4	40.2 (0.5)	1.28	0.73–2.27	1.19	0.68–2.08	0.930	0.54–1.60	
				*p* = 0.443							
Saturated fat (g/day)											0.842
Food security	14	12	4, 24	72.2 (66.8)	1.0		1.0		1.0		
Low food security	13	10	3, 23	72.3 (4.6)	0.96	0.76–1.22	1.04	0.81–1.34	1.13	0.87–1.45	
Very low food security	9	8	2, 20	74.7 (0.9)	0.79	0.40–1.54	0.93	0.44–1.95	1.05	0.48–2.31	
				*p* = 0.717							
Dietary cholesterol (mg/day)											0.185
Food security	262	208	37, 538	69.4 (64.2)	1.0		1.0		1.0		
Low food security	253	170	26, 590	70.6 (4.5)	0.90	0.70–1.15	1.04	0.79–1.36	1.29	0.97–1.72	
Very low food security	202	96	8, 493	80.5 (1.0)	0.73	0.41–1.31	1.20	0.66–2.20	1.73	0.95–3.16	
				*p* = 0.405							
Protein (g/day)											0.021
Food security	73	67	35, 111	64.8 (60.0)	1.0		1.0		1.0		
Low food security	66	62	29, 104	55.3 (3.5)	1.49 **	1.19–1.85	1.32	0.97–1.80	0.99	0.72–1.37	
Very low food security	52	44	25, 93	37.9 (0.5)	3.06 ***	1.84–5.08	1.72	0.72–4.10	1.17	0.51–2.68	
				*p* < 0.001							
Dietary fiber (g/day)											0.744
Food security	24	21	11, 40	48.4 (44.7)	1.0		1.0		1.0		
Low food security	21	20	9, 37	46.9 (3.0)	1.07	0.85–1.35	1.02	0.79–1.32	0.86	0.66–1.12	
Very low food security	19	19	6, 35	37.9 (0.5)	1.65	0.97–2.80	1.01	0.52–1.94	0.79	0.41–1.55	
				*p* = 0.149							
Vitamin A (μgRE/day)											0.403
Food security	729	536	196, 1333	33.8 (31.2)	1.0		1.0		1.0		
Low food security	734	486	165, 1390	31.6 (2.0)	0.99	0.77–1.28	0.932	0.71–1.22	0.83	0.63–1.09	
Very low food security	662	408	109, 1422	24.1 (0.3)	1.24	0.71–2.18	0.855	0.47–1.55	0.71	0.39–1.29	
				*p* = 0.752							
Thiamin (mg/day)											0.072
Food security	2.1	1.9	1.0, 3.1	86.6 (80.0)	1.0		1.0		1.0		
Low food security	2.0	1.8	0.9, 3.0	81.9 (5.2)	1.41 *	1.05–1.90	1.12	0.80–1.57	0.99	0.69–1.42	
Very low food security	1.6	1.4	0.7, 2.8	73.6 (0.9)	2.61 **	1.55–4.39	1.19	0.57–2.46	0.94	0.46–1.91	
				*p* < 0.001							
Riboflavin (mg/day)											0.841
Food security	1.4	1.3	0.6, 2.3	45.0 (41.6)	1.0		1.0		1.0		
Low food security	1.3	1.2	0.5, 2.0	40.0 (2.5)	1.24	0.98–1.55	1.06	0.81–1.40	0.84	0.63–1.12	
Very low food security	1.1	0.8	0.3, 2.2	27.6 (0.3)	1.99 *	1.14–3.47	0.97	0.48–1.95	0.66	0.33–1.32	
				*p* = 0.009							
Niacin (mg/day)											0.406
Food security	17	15	8, 27	48.0 (44.4)	1.0		1.0		1.0		
Low food security	15	14	7, 24	43.1 (2.7)	1.24 *	1.00–1.54	1.10	0.88–1.37	0.91	0.72–1.14	
Very low food security	12	11	5, 22	28.7 (0.3)	2.26 **	1.30–3.92	1.32	0.71–2.47	0.95	0.51–1.75	
				*p* = 0.002							
Vitamin C (mg/day)											0.080
Food security	98	59	18, 235	33.9 (31.3)	1.0		1.0		1.0		
Low food security	84	50	15, 198	27.0 (1.7)	1.37 *	1.07–1.74	1.38 *	1.07–1.78	1.14	0.87–1.48	
Very low food security	58	34	9, 136	21.8 (0.3)	2.17 *	1.12–4.21	1.80	0.87–3.71	1.32	0.65–2.71	
				*p* = 0.003							
Calcium (mg/day)											0.306
Food security	498	442	215, 822	13.4 (12.4)	1.0		1.0		1.0		
Low food security	463	406	199, 765	11.1 (0.7)	1.19	0.83–1.69	1.09	0.75–1.57	0.90	0.61–1.33	
Very low food security	368	378	127, 631	3.4 (0.0)	4.75 **	1.48–15.25	3.11	0.88–10.91	2.35	0.68–8.12	
				*p* = 0.022							
Phosphorus (mg/day)											0.002
Food security	1103	1047	568, 1655	80.4 (74.3)	1.0		1.0		1.0		
Low food security	1010	942	514, 1530	73.9 (4.7)	1.47 **	1.15–1.88	1.19	0.88–1.62	0.88	0.63–1.23	
Very low food security	828	759	387, 1273	56.3 (0.7)	4.10 ***	2.51–6.68	3.12*	1.07–9.12	1.81	0.65–5.02	
				*p* < 0.001							
Sodium (mg/day)											0.736
Food security	4055	3608	1573, 6713	8.8(8.1)	1.0		1.0		1.0		
Low food security	3776	3384	1426, 6171	9.5(0.6)	1.07	0.73–1.56	1.31	0.89–1.94	1.38	0.92–2.07	
Very low food security	3151	2747	1151, 5911	17.2(0.2)	0.61	0.31–1.21	1.03	0.52–2.05	0.97	0.50–1.88	
				*p* = 0.331							
Potassium (mg/day)											0.278
Food security	3072	2830	1534, 4772	30.2(27.9)	1.0		1.0		1.0		
Low food security	2708	2470	1304, 4322	24.6(1.6)	1.39 *	1.08–1.78	1.30	0.95–1.79	1.01	0.72–1.40	
Very low food security	2251	2217	1015, 3696	17.2(0.2)	2.38 **	1.31–4.32	1.27	0.61–2.65	0.90	0.43–1.88	
				*p* = 0.009							
Iron (mg/day)											0.106
Food security	17	15	8, 28	72.1(66.7)	1.0		1.0		1.0		
Low food security	16	14	7, 28	69.5(4.4)	1.16	0.92–1.46	0.98	0.75–1.28	0.90	0.68–1.19	
Very low food security	14	12	5, 24	59.8(0.7)	2.02 **	1.24–3.29	1.58	0.78–3.18	1.40	0.72–2.75	
				*p* = 0.009							

KDRIs, Dietary Reference Intakes for Koreans. ^1^ % meeting guideline: by food security group % (by total %). ^2^ Model 1: unadjusted. ^3^ Model 2: adjusted for age, sex, survey year, total energy intake. ^4^ Model 3: adjusted for age, sex, survey year, total energy intake, household size, education, marital status, household income level, physical activity level. * *p* < 0.05, ** *p* < 0.01, *** *p* < 0.001.

**Table 3:** Difference in means of KHEI item score according to household food security status.

	Median	10th, 90th Percentile	Mean ± SE ^1^	*p*-Value ^1^
Mixed grain intake				0.153
Food security	1.7	0, 5.0	2.2 ± 0.04	
Low food security	1.0	0, 5.0	2.0 ± 0.13	
Very low food security	0	0, 5.0	1.8 ± 0.24	
Total fruit intake				0.195
Food security	1.6	0, 5.0	2.2 ± 0.04	
Low food security	0.3	0, 5.0	1.8 ± 0.11	
Very low food security	0	0, 5.0	1.4 ± 0.24	
Fresh fruit intake				0.081
Food security	2.1	0, 5.0	2.4 ± 0.04	
Low food security	0	0, 5.0	2.0 ± 0.13	
Very low food security	0	0, 5.0	1.4 ± 0.27	
Total vegetable intake				0.822
Food security	3.9	1.5, 5.0	3.6 ± 0.02	
Low food security	3.5	1.3, 5.0	3.4 ± 0.09	
Very low food security	3.5	1.0, 5.0	3.2 ± 0.19	
Vegetable intake excluding kimchi and pickled vegetables				0.528
Food security	3.5	1.0, 5.0	3.3 ± 0.02	
Low food security	3.0	0.7, 5.0	3.1 ± 0.09	
Very low food security	3.0	0.3, 5.0	2.9 ± 0.23	
Meat/fish/egg/legume intake				0.141
Food security	7.8	2.4, 10.0	7.1 ± 0.05	
Low food security	7.4	1.6, 10.0	6.7 ± 0.19	
Very low food security	5.3	0.4, 10.0	5.3 ± 0.43	
Milk and dairy products intake				0.583
Food security	0	0, 10.0	3.3 ± 0.07	
Low food security	0	0, 10.0	2.8 ± 0.24	
Very low food security	0	0, 10.0	2.2 ± 0.48	
Having breakfast				<0.001
Food security	6.5	0, 10.0	6.9 ± 0.07 ^a^	
Low food security	6.1	0, 10.0	6.4 ± 0.25 ^ab^	
Very low food security	4.5	0, 10.0	5.6 ± 0.53 ^b^	
% of energy from saturated fatty acid				0.411
Food security	10.0	0, 10.0	7.8 ± 0.06	
Low food security	9.9	0, 10.0	7.8 ± 0.19	
Very low food security	10.0	0, 10.0	8.1 ± 0.42	
Sodium intake				0.650
Food security	6.4	0, 10.0	5.8 ± 0.05	
Low food security	6.9	0.7, 10.0	6.2 ± 0.17	
Very low food security	8.2	1.3, 10.0	7.0 ± 0.37	
% of energy from sweets and beverages				0.080
Food security	10.0	6.0, 10.0	9.0 ± 0.04	
Low food security	9.9	3.7, 10.0	8.8 ± 0.17	
Very low food security	9.7	0, 10.0	8.2 ± 0.37	
% of energy from carbohydrate				0.110
Food security	2.9	0, 5.0	2.7 ± 0.03	
Low food security	3.0	0, 5.0	2.6 ± 0.12	
Very low food security	0.7	0, 5.0	2.0 ± 0.25	
% of energy from fat				0.020
Food security	5.0	0, 5.0	3.5 ± 0.03	
Low food security	5.0	0, 5.0	3.6 ± 0.11	
Very low food security	4.1	0, 5.0	2.8 ± 0.25	
Total energy intake				0.097
Food security	5.0	0, 5.0	3.4 ± 0.03	
Low food security	5.0	0, 5.0	3.2 ± 0.12	
Very low food security	3.8	0, 5.0	2.7 ± 0.27	
Total score				<0.001
Food security	63.9	47.4, 80.1	63.2 ± 0.22 ^a^	
Low food security	60.6	44.6, 77.8	60.3 ± 0.71 ^ab^	
Very low food security	55.4	37.2, 71.2	54.6 ± 1.66 ^b^	

KHEI, Korean Healthy Eating Index. ^1^ Tukey’s test of multiple comparison and *p*-value were adjusted for age, sex, survey year, total energy intake, household size, education, marital status, household income level, physical activity level. ^a, ab, b^ Different letters in the same column indicate the significant statistical difference, same letters indicate no significant difference (*p* < 0.05, Tukey’s test).

**Table 4:** Dietary quality measured by the KHEI according to household food security status.

	% Full Score of KHEI ^1^	Less than Full Score						% Zero Score of KHEI ^2^	Zero Score					
		Model 1 ^3^		Model 2 ^4^		Model 3 ^5^			Model 1 ^3^		Model 2 ^4^		Model 3 ^5^	
		OR	95% CI	OR	95% CI	OR	95% CI		OR	95% CI	OR	95% CI	OR	95% CI
Mixed grain intake	(30.9)							(38.5)						
Food security	31.1 (28.8)	1.0		1.0		1.0		38.3 (35.4)	1.0		1.0		1.0	
Low food security	29.6 (1.9)	1.21	0.93–1.57	1.22	0.94–1.59	1.18	0.90–1.56	38.7 (2.4)	1.08	0.84–1.38	1.11	0.86–1.44	1.06	0.80–1.39
Very low food security	19.5 (0.2)	1.73	0.92–3.24	1.71	0.91–3.20	1.69	0.88–3.21	48.3 (0.6)	1.50	0.92–2.44	1.54	0.93–2.52	1.39	0.83–2.34
	*p =* 0.093	*p trend* = 0.016						*p =* 0.265	*p trend* = 0.106					
Total fruit intake	(29.6)							(31.8)						
Food security	30.2 (27.9)	1.0		1.0		1.0		31.1 (28.8)	1.0		1.0		1.0	
Low food security	22.8 (1.4)	1.54 **	1.18–2.00	1.65 **	1.26–2.16	1.32	1.00–1.75	38.1 (2.4)	1.38 *	1.07–1.78	1.43 **	1.10–1.86	1.07	0.82–1.40
Very low food security	19.5 (0.2)	1.66	0.90–3.05	1.42	0.74–2.71	0.94	0.48–1.83	52.9 (0.6)	2.56 **	1.53–4.29	2.35 **	1.34–4.10	1.49	0.86–2.58
	*p =* 0.002	*p trend* = 0.039						*p* < 0.001	*p trend* = 0.059					
Fresh fruit intake	(42.7)							(42.8)						
Food security	43.3 (40.1)	1.0		1.0		1.0		42.0 (38.8)	1.0		1.0		1.0	
Low food security	36.5 (2.3)	1.36 *	1.07–1.74	1.47 **	1.14–1.89	1.18	0.91–1.54	50.0 (3.2)	1.39 **	1.10–1.76	1.49 **	1.16–1.91	1.16	0.89–1.49
Very low food security	25.3 (0.3)	2.04 *	1.12–3.72	1.88	0.98–3.61	1.34	0.68–2.61	64.4 (0.8)	2.34 **	1.41–3.86	2.28 **	1.30–3.97	1.55	0.88–2.73
	*p =* 0.003	*p trend* = 0.043						*p* < 0.001	*p trend =* 0.020					
Total vegetable intake	(34.2)							(0.4)						
Food security	34.5 (31.9)	1.0		1.0		1.0		0.4 (0.4)	1.0		1.0		1.0	
Low food security	30.5 (1.9)	1.22	0.97–1.55	1.14	0.88–1.48	1.08	0.83–1.42	0.7 (0.0)	1.82	0.48–6.91	1.70	0.39–7.37	0.99	0.17–5.67
Very low food security	27.6 (0.3)	1.33	0.73–2.40	0.95	0.49–1.86	0.87	0.45–1.65	2.3 (0.0)	2.45	0.57–10.50	1.54	0.27–8.70	0.92	0.14–6.23
	*p =* 0.184	*p trend* = 0.465						*p =* 0.397	*p trend* = 0.645					
Vegetable intake excluding kimchi and pickled vegetables	(31.1)							(1.4)						
Food security	31.5 (29.1)	1.0		1.0		1.0		1.3 (1.2)	1.0		1.0		1.0	
Low food security	25.2 (1.6)	1.34 *	1.03–1.73	1.27	0.96–1.67	1.14	0.86–1.51	1.8 (0.1)	1.14	0.46–2.88	1.04	0.39–2.77	0.66	0.21–2.07
Very low food security	28.7 (0.3)	1.11	0.62–1.99	0.81	0.43–1.52	0.69	0.36–1.30	3.4 (0.0)	2.77	0.65–11.92	1.88	0.34–10.37	0.99	0.21–4.71
	*p =* 0.094	*p trend* = 0.739						*p =* 0.328	*p trend* = 0.799					
Meat/fish/egg/legume intake	(34.7)							(0.5)						
Food security	35.3 (32.6)	1.0		1.0		1.0		0.4 (0.4)	1.0		1.0		1.0	
Low food security	29.0 (1.8)	1.27	1.00–1.61	1.12	0.84–1.49	0.95	0.70–1.28	1.3 (0.1)	3.15 *	1.09–9.16	2.96	0.94–9.35	1.98	0.53–7.38
Very low food security	18.4 (0.2)	2.79 **	1.53–5.09	1.67	0.80–3.49	1.37	0.65–2.91	3.4 (0.0)	10.11 **	2.21–46.33	6.52 *	1.13–37.53	3.39	0.90–12.80
	*p =* 0.001	*p trend* = 0.123						*p* < 0.001	*p trend* = 0.054					
Milk and dairy products intake	(23.7)							(59.0)						
Food security	24.0 (22.2)	1.0		1.0		1.0		58.5 (54.1)	1.0		1.0		1.0	
Low food security	20.8 (1.3)	1.20	0.92–1.56	1.16	0.89–1.51	1.00	0.76–1.31	64.2 (4.1)	1.35 *	1.05–1.74	1.34 *	1.04–1.73	1.16	0.89–1.51
Very low food security	19.5 (0.2)	1.64	0.89–3.00	1.40	0.77–2.56	1.07	0.59–1.95	71.3 (0.9)	2.06 **	1.21–3.49	1.83 *	1.07–3.12	1.41	0.83–2.39
	*p =* 0.119	*p trend* = 0.491						*p =* 0.002	*p trend* = 0.049					
Having breakfast	(60.9)							(14.6)						
Food security	61.5 (56.9)	1.0		1.0		1.0		14.3 (13.2)	1.0		1.0		1.0	
Low food security	56.2 (3.6)	1.23	0.99–1.54	1.35 *	1.06–1.72	1.39 *	1.08–1.80	16.6 (1.0)	1.44 *	1.07–1.96	1.51 **	1.12–2.03	1.48 *	1.10–2.00
Very low food security	41.4 (0.5)	2.06*	1.17–3.63	2.35 **	1.27–4.36	2.59 **	1.35–4.99	29. 9 (0.4)	1.77 *	1.07–2.93	1.72	0.99–2.96	1.68	0.89–3.18
	*p =* 0.007	*p trend* < 0.001						*p =* 0.005	*p trend* < 0.001					
% of energy from saturated fatty acid	(68.9)							(10.4)						
Food security	68.8 (63.6)	1.0		1.0		1.0		10.3 (9.5)	1.0		1.0		1.0	
Low food security	69.7 (4.4)	0.95	0.76–1.20	1.05	0.82–1.33	1.12	0.88–1.43	10.6 (0.7)	0.99	0.71–1.38	1.09	0.78–1.54	1.15	0.82–1.63
Very low food security	67.8 (0.8)	0.95	0.50–1.77	1.21	0.59–2.46	1.33	0.63–2.81	13.8 (0.2)	1.20	0.65–2.22	1.53	0.77–3.03	1.61	0.79–3.30
	*p =* 0.919	*p trend* = 0.484						*p =* 0.862	*p trend* = 0.331					
Sodium intake	(18.1)							(11.2)						
Food security	17.6 (16.3)	1.0		1.0		1.0		11.4 (10.5)	1.0		1.0		1.0	
Low food security	21.7 (1.4)	0.89	0.68–1.17	1.19	0.87–1.62	1.42 *	1.02–1.98	9.3 (0.6)	0.83	0.59–1.19	0.96	0.64–1.45	0.98	0.64–1.50
Very low food security	37.9 (0.5)	0.32 ***	0.20–0.53	0.54	0.29–1.03	0.70	0.38–1.28	6.9 (0.1)	0.557	0.25–1.26	1.01	0.39–2.62	1.06	0.41–2.75
	*p* < 0.001	*p trend* = 0.153						*p =* 0.239	*p trend* = 0.607					
% of energy from sweets and beverages	(79.6)							(3.6)						
Food security	79.8 (73.8)	1.0		1.0		1.0		3.3 (3.1)	1.0		1.0		1.0	
Low food security	77.9 (4.9)	1.01	0.78–1.31	0.99	0.76–1.30	1.00	0.76–1.31	6.0 (0.4)	1.58	0.98–2.52	1.55	0.95–2.52	1.55	0.95–2.52
Very low food security	70.1 (0.9)	1.60	0.96–2.67	1.45	0.87–2.43	1.38	0.82–2.33	10.3 (0.1)	3.21 **	1.55–6.61	2.84 **	1.45–5.55	2.73 **	1.32–5.65
	*p =* 0.192	*p trend* = 0.204						*p =* 0.001	*p trend* = 0.002					
% of energy from carbohydrate	(29.0)							(27.2)						
Food security	29.3 (27.1)	1.0		1.0		1.0		26.8 (24.7)	1.0		1.0		1.0	
Low food security	25.7 (1.6)	1.20	0.94–1.54	1.16	0.91–1.49	1.04	0.80–1.32	30.3 (1.9)	1.09	0.84–1.41	1.05	0.80–1.36	0.89	0.68–1.17
Very low food security	24.1 (0.3)	1.21	0.69–2.11	1.09	0.63–1.90	0.90	0.51–1.60	43.7 (0.5)	2.28 **	1.46–3.56	2.14 **	1.38–3.31	1.64 *	1.04–2.57
	*p =* 0.290	*p trend* = 0.891						*p =* 0.003	*p trend* = 0.264					
% of energy from fat	(55.3)							(18.6)						
Food security	55.5 (51.4)	1.0		1.0		1.0		18.3 (16.9)	1.0		1.0		1.0	
Low food security	54.2 (3.4)	1.02	0.80–1.29	0.99	0.78–1.26	0.87	0.67–1.13	20.1 (1.3)	0.91	0.69–1.19	0.88	0.67–1.15	0.72 *	0.54–0.97
Very low food security	43.7 (0.5)	1.59	0.98–2.59	1.50	0.94–2.40	1.21	0.75–1.96	36.8 (0.4)	2.82 ***	1.80–4.42	2.68 ***	1.73–4.13	1.93 **	1.23–3.02
	*p =* 0.178	*p trend* = 0.772						*p* < 0.001	*p trend* = 0.589					
Total energy intake	(55.1)							(21.7)						
Food security	55.4 (51.3)	1.0		1.0		1.0		21.3 (19.7)	1.0		1.0		1.0	
Low food security	52.4 (3.3)	1.11	0.90–1.38	1.12	0.90–1.38	1.06	0.84–1.32	24.8 (1.6)	1.29	0.99–1.69	1.30	0.99–1.70	1.20	0.91–1.59
Very low food security	43.7 (0.5)	1.47	0.92–2.36	1.49	0.93–2.39	1.32	0.82–2.14	33.3 (0.4)	1.84 *	1.09–3.12	1.87 *	1.11–3.13	1.59	0.92–2.74
	*p =* 0.178	*p trend* = 0.205						*p =* 0.015	*p trend* = 0.033					

KHEI, Korean Healthy Eating Index. ^1^ Percentage of people with the highest scores on KHEI items. ^2^ Percentage of people with a score of zero on KHEI items. ^1,2^ By food security group % (by total %). ^3^ Model 1: unadjusted. ^4^ Model 2: adjusted for age, sex, survey year, total energy intake. ^5^ Model 3: adjusted for age, sex, survey year, total energy intake, household size, education, marital status, household income level, physical activity level. * *p* < 0.05, ** *p* < 0.01, *** *p* < 0.001.

## Data Availability

The data are available from the Korean National Health and Nutrition Examination Survey (KNHANES) website.

## References

[1] Afshin A., Sur P.J., Fay K.A., Cornaby L., Ferrara G., Salama J.S., Mullany E.C., Abate K.H., Abbafati C., GBD 2017 Diet Collaborators (2019). Health effects of dietary risks in 195 countries, 1990–2017: A systematic analysis for the Global Burden of Disease Study 2017. Lancet.

[2] Leung C.W., Epel E.S., Ritchie L.D., Crawford P.B., Laraia B.A. (2014). Food insecurity is inversely associated with diet quality of lower-income adults. J. Acad. Nutr. Diet..

[3] Kim H.R. (2013). Nutrition transition and shiting diet linked noncommunicable diseases and policy issues. Health Welf Policy Forum.

[4] Guthrie J.F., Nord M. (2002). Federal activities to monitor food security. J. Acad. Nutr. Diet..

[5] Champagne C.M., Casey P.H., Connell C.L., Stuff J.E., Gossett J.M., Harsha D.W., McCabe-Sellers B., Robbins J.M., Simpson P.M., Weber J.L. (2007). Poverty and food intake in rural America: Diet quality is lower in food insecure adults in the Mississippi Delta. J. Am. Diet. Assoc..

[6] Pei C.S., Appannah G., Sulaiman N. (2018). Household food insecurity, diet quality, and weight status among indigenous women (Mah Meri) in Peninsular Malaysia. Nutr. Res. Pract..

[7] Rodríguez L.A., Mundo-Rosas V., Méndez-Gómez-Humarán I., Pérez-Escamilla R., Shamah-Levy T. (2017). Dietary quality and household food insecurity among Mexican children and adolescents. Matern. Child Nutr..

[8] Huet C., Rosol R., Egeland G.M. (2012). The prevalence of food insecurity is high and the diet quality poor in Inuit communities. J. Nutr..

[9] Tarasuk V.S., Beaton G.H. (1999). Women’s dietary intakes in the context of household food insecurity. J. Nutr..

[10] Kirkpatrick S.I., Tarasuk V. (2008). Food insecurity is associated with nutrient inadequacies among Canadian adults and adolescents. J. Nutr..

[11] Mclaughlin C., Tarasuk V., Kreiger N. (2003). An examination of at-home food preparation activity among low-income, food-insecure women. J. Am. Diet. Assoc..

[12] Olson C.M. (1999). Symposium: Advances in Measuring Food Insecurity and Hunger in the US Introduction. J. Nutr..

[13] Yang Y.J. (2015). Socio-demographic characteristics, nutrient intakes and mental health status of older Korean adults depending on household food security: Based on the 2008-2010 Korea National Health and Nutrition Examination Survey. Korean J. Commun. Nutr..

[14] Jun S., Hong E., Joung H. (2015). Flavonoid intake according to food security in Korean adults: Based on the Korea National Health and Nutrition Examination Survey 2007–2012. J. Nutr. Health.

[15] Lane G., Nisbet C., Vatanparast H. (2019). Food Insecurity and Nutritional Risk among Canadian Newcomer Children in Saskatchewan. Nutrients.

[16] Kim H.J., Oh K. (2015). Household food insecurity and dietary intake in Korea: Results from the 2012 Korea National Health and Nutrition Examination Survey. Public Health Nutr..

[17] Shim J.S., Oh K.W., Nam C.M. (2008). Association of Household Food Security with Dietary Intake; Based on the Third (2005) Korea National Health and Nutrition Examination Survey (KNHANES III). J. Nutr. Health.

[18] Lim H.S., Park Y.H., Lee H.H., Kim T.H., Kim S.K. (2015). Comparison of calcium intake status by region and socioeconomic status in Korea: The 2011-2013 Korea National Health and Nutrition Examination Survey. J. Bone Metab..

[19] Hur I., Jang M.J., Oh K. (2011). Food and nutrient intakes according to income in Korean men and women. Osong Public Health Res. Perspect..

[20] Shim J.E., Yoon J., Lee K., Kwon S. (2009). Evaluation of dietary intake of Korean school-aged children from low-income families by comparing with the Korean food guide: Analysis of the data from the 2001 National Health and Nutrition Survey. Korean J. Nutr..

[21] Chun I., Ryu S.Y., Park J., Ro H.K., Han M.A. (2015). Associations between food insecurity and healthy behaviors among Korean adults. Nutr. Res. Pract..

[22] Lee S.E., Song Y.J., Kim Y., Choe J., Paik H.Y. (2016). Household food insufficiency is associated with dietary intake in Korean adults. Public Health Nutr..

[23] Chung H.K., Kim O.Y., Kwak S.Y., Cho Y., Lee K.W., Shin M.J. (2016). Household food insecurity is associated with adverse mental health indicators and lower quality of life among Koreans: Results from the Korea National Health and Nutrition Examination Survey 2012–2013. Nutrients.

[24] Oh S.Y., Hong M.J. (2003). Food insecurity is associated with dietary intake and body size of Korean children from low-income families in urban areas. Eur. J. Clin. Nutr..

[25] Kwon S.O., Oh S.Y. (2007). Associations of household food insecurity with socioeconomic measures, health status and nutrient intake in low income elderly. J. Nutr. Health.

[26] Lee K., Yoo H.S. (2016). Association of food insecurity and depression in Korean adults. JKAIS.

[27] Kim K., Kim M.K., Shin Y.J. (2009). Household food insecurity and its characteristics in Korea. Health Soc. Welf Rev..

[28] Trijsburg L., Talsma E.F., De Vries J.H., Kennedy G., Kuijsten A., Brouwer I.D. (2019). Diet quality indices for research in low-and middle-income countries: A systematic review. Nutr. Rev..

[29] Guenther P.M., Reedy J., Krebs-Smith S.M., Reeve B.B., Basiotis P.P. (2007). Development and Evaluation of the Healthy Eating Index-2005.

[30] Krebs-Smith S.M., Pannucci T.E., Subar A.F., Kirkpatrick S.I., Lerman J.L., Tooze J.A., Wilson M.M., Reedy J. (2018). Update of the healthy eating index: HEI-2015. J. Acad. Nutr. Diet..

[31] Woodruff S.J., Hanning R.M. (2010). Development and implications of a revised Canadian healthy eating index (HEIC-2009). Public Health Nutr..

[32] Hiza H.A., Casavale K.O., Guenther P.M., Davis C.A. (2013). Diet quality of Americans differs by age, sex, race/ethnicity, income, and education level. J. Acad. Nutr. Diet..

[33] Korea Centers for Disease Control and Prevention (2016). Korea Health Statistics 2015: Korea National Health and Nutrition Examination Survey (KNHANES VI-3).

[34] English site for Food Composition Database. http://koreanfood.rda.go.kr/eng/fctFoodSrchEng/main.

[35] Yook S.M., Park S., Moon H.K., Kim K., Shim J.E., Hwang J.Y. (2015). Development of Korean healthy eating index for adults using the Korea national health and nutrition examination survey data. J. Nutr. Health.

[36] Yun S., Oh K. (2018). Development and status of Korean Healthy Eating Index for adults based on the Korea National Health and Nutrition Examination Survey. Public Health Wkly Rep..

[37] Banna J.C., McCrory M.A., Fialkowski M.K., Boushey C. (2017). Examining plausibility of self-reported energy intake data: Considerations for method selection. Front. Nutr..

[38] Korea Centers for Disease Control and Prevention (2019). The Sixth Korea National Health and Nutrition Examination Survey (KNHANES VI-3).

[39] The Ministry of Health and Welfare (2015). The Korean Nutrition Society. Dietary Reference Intakes for Koreans 2015.

[40] Tugault-Lafleur C.N., Black J.L., Barr S.I. (2017). A systematic review of methods to assess children’s diets in the school context. Adv. Nutr..

[41] Lynch S.R. (2011). Why nutritional iron deficiency persists as a worldwide problem. J. Nutr..

[42] Workicho A., Belachew T., Feyissa G.T., Wondafrash B., Lachat C., Verstraeten R., Kolsteren P. (2016). Household dietary diversity and Animal Source Food consumption in Ethiopia: Evidence from the 2011 Welfare Monitoring Survey. BMC Public Health.

[43] Lee Y.S., Kim T.H. (2019). Household food insecurity and breakfast skipping: Their association with depressive symptoms. Psychiatry Res..

[44] Crews D.C., Kuczmarski M.F., Grubbs V., Hedgeman E., Shahinian V.B., Evans M.K., Zonderman A.B., Burrows N.R., Williams D.E., Saran R. (2014). Effect of food insecurity on chronic kidney disease in lower-income Americans. Am. J. Nephrol..

[45] Kim D.W., Lee M.S., Na B.J., Hong J.Y. (2013). Health-related dietary behaviors and lifestyle factors associated with sodium hyperingestion in Korean adults. JKAIS.

[46] Kim J.H., Lim G.E., Kang S., Lee K., Park T.J., Kim J. (2015). The relationship between daily sodium intake and obesity in Korean adults. Korean J. Health Promot..

[47] Kim N., Kim G.U., Kim H. (2020). Comparative Study of Dietary Patterns by Living Arrangements: The Korea National Health and Nutrition Examination Survey (KNHANES) 2013–2015. Int. J. Environ. Res. Public Health.

[48] Shim J.E., Paik H.Y., Moon H.K. (2007). Breakfast consumption pattern, diet quality and health outcomes in adults from 2001 National Health and Nutrition Survey. J. Nutr. Health.

[49] Tarasuk V.S. (2001). Household food insecurity with hunger is associated with women’s food intakes, health and household circumstances. J. Nutr..

[50] Lee S.H., Chung S.J., Choi K.R. (2011). Relationship between nutrient intake and biochemical index with breakfast eating in Korean adults: Analysis of data from the 2007 National Health and Nutrition Survey. Korean J. Food Cult..

[51] Gundersen C., Kreider B., Pepper J. (2011). The economics of food insecurity in the United States. Appl. Econ. Perspect. Policy.

[52] Lee J., Shin A. (2015). Vegetable and fruit intake in one person household: The Korean National Health and Nutrition Examination Survey (2010–2012). J. Nutr. Health.

[53] Kendall A., Olson C.M., Frongillo E.A. (1996). Relationship of hunger and food insecurity to food availability and consumption. J. Am. Diet. Assoc..

[54] Bawadi H.A., Tayyem R.F., Dwairy A.N., Al-Akour N. (2012). Prevalence of food insecurity among women in northern Jordan. J. Health Popul. Nutr..

[55] Taylor C.A., Spees C.K., Markwordt A.M., Watowicz R.P., Clark J.K., Hooker N.H. (2017). Differences in US adult dietary patterns by food security status. J. Consum. Aff..

[56] Lee J.S., Kim H.Y., Hwang J.Y., Kwon S., Chung H.R., Kwak T.K., Kang M.H., Choi Y.S. (2018). Development of Nutrition Quotient for Korean adults: Item selection and validation of factor structure. J. Nutr. Health.

[57] Jang H.B., Park J.Y., Lee H.J., Kang J.H., Park K.H., Song J. (2011). Association between parental socioeconomic level, overweight, and eating habits with diet quality in Korean sixth grade school children. Korean J. Nutr..

[58] Alaimo K., Briefel R.R., Frongillo E.A., Olson C.M. (1998). Food insufficiency exists in the United States: Results from the third National Health and Nutrition Examination Survey (NHANES III). Am. J. Public Health.

[59] Wolfson J.A., Leung C.W. (2020). Food insecurity and COVID-19: Disparities in early effects for US adults. Nutrients.

